# Role of local town planning authorities in building collapse in Nigeria: evidence from Enugu metropolis

**DOI:** 10.1016/j.heliyon.2020.e04361

**Published:** 2020-07-09

**Authors:** Francis O. Okeke, Chinwe G. Sam-amobi, Francis I. Okeke

**Affiliations:** a Department of Architecture, University of Nigeria, Enugu Campus, Enugu State, Nigeria; b Department of Geo-informatics and Survey, University of Nigeria, Enugu Campus, Enugu State, Nigeria

**Keywords:** Building professionals, Building collapse, Planning approval, Building legislation, Enugu metropolis, Civil engineering, Environmental science, Environmental engineering, Environmental Impact Assessment, Arts and humanities, Architecture

## Abstract

The current spate of building collapse in Nigeria has continued to attract research efforts to unravel the causes and possible remedies. Although cases of building collapse in Nigeria has been associated with several factors, those factors associated with building plan approval process have not adequately investigated, especially in a rapidly expanding colonial city of Enugu. The study investigated the role of local town planning authorities in the increasing cases of collapsed buildings in Nigeria using Enugu as a case study. A well-structured questionnaire was administered to the three Chief Town Planners in the three planning approval offices and oral interviews randomly selected 30 developers in ongoing construction projects within Enugu metropolis were conducted. Using content analysis and descriptive statistics the data collected were analyzed. It was observed that due to poor staffing and lack of engagement of building professionals, the planning approval authorities were not effective in scrutinizing, vetting and evaluating building drawings submitted for approval as well as in supervising and monitoring the level of compliance of buildings under construction with the operational building codes and bye-laws in the study area. The study concludes that these lapses in the roles of local building approval authorities can contribute to the increasing cases of collapsed buildings in Enugu Metropolis. It recommends that government should take proactive steps by engaging the right number of building professionals in her planning approval offices and ensuring strict enforcement of the existing physical development legislation and punishment of offenders.

## 1 Introduction

In spite of the revolution in architectural design and building construction in the 21st century, which was brought about by the advancement in structural engineering, there are still incidences of building failure around the globe and particularly in countries in sub-Saharan Africa. Evidence in the literature indicate that the rate of building collapse is increasing in many developing countries [1] as well as awareness of the devastating effects this has on the social and environmental fabrics of cities around the world [2]. However, the pertinent issue is not only the after effects of building collapse but the gamut of factors that contribute to its occurrence. Evidence in the literature suggests that while building collapse has been linked to inferno, acts of terrorism, global changes in the environment and seismic activities in many western countries [3], in countries in sub-Saharan Africa, most cases of building collapse have been attributed mainly to human factors such design errors and negligence, and lack of institutional mechanisms and capacity to prevent the collapse of buildings [2, 4]. It was on this premise that some authors have argued that to curb the growing incidences of building collapse in countries in sub-Saharan Africa, there was a need to re-examine the entire process and procedures involved in building design, construction and management to identify the underlying factors that precipitate this challenge [5].

Previous studies have revealed that scholarship in the global North on failure of building is largely dominated by engineers (structural, electrical and mechanical) with emphasis on experimental research design coupled with structural computational analysis and statistical modelling and forensic scientists whose analyses are difficult to understand from the perspective of the social sciences [6, 7]. Although, in Africa some studies on building collapse have been done in countries like Kenya, Ghana, Ugandan, South Africa and Cameron [8, 9, 10, 11], the review of published literature reveals that Nigerian scholars seem to have taken the lead in research on this subject. This is probably because building failure has turn out to be a regular occurrence in this country. Buildings are products of inputs from diverse experts in the fields of architecture, engineering, quantity survey and planning, and as a result multidisciplinary approaches have been applied in investigating and understanding the causes and remedies for building collapse. Notably, in building design, architects establish realities once constructed are not altered easily [12], and therefore, any building once properly designed and constructed is expected to be useable throughout its life span provided established quality parameters are used in the construction process and appropriate maintenance practices are engaged at the operational stage of the building. It is however understood that when buildings are subjected to abnormal loads that are far and above the design loads, they may suffer extensive damage [13, 14], which in some cases might lead to collapse and causalities.

In a developing country like Nigeria, the review of literature and anecdotal evidence reveal that between 1974 and 2019, which is 45 years, over 221 incidences of collapsed buildings have been reported in her major towns and cities leading to several fatalities. In fact, within the last four years, Nigeria has recorded over 56 cases of building collapse [15]. There is no state in Nigeria, without at least one incident of building collapse in the past ten years [16]. Available records also show that between 1971 and 2016, 1,455 fatalities were recorded in 175 collapse cases [17]. The Nigeria Construction and Infrastructure Summit Group estimates that the country loses between 2.03tn and 3.05tn annually to infrastructure deficit from building failure because the magnitude of overall damage to the initial cause is usually out of proportion due to progressive collapse. Resulting from these, research efforts have been focused on identifying the various factors that contribute to the burgeoning cases of collapsed buildings in this country through physical observations and or sample collection of debris from building collapse sites and oral interviews of eye witnesses or residents within the vicinities. Among the factors identified by the existing studies include the lack of soil test investigation, poor design, dysfunctional construction, lack of adherence to established building standards, lack of enforcement of building codes/regulations/bye-laws, use of substandard construction materials, engagement of non-professionals and poorly trained workmen, poor supervision, excessive loading of buildings, poor maintenance practices, heavy downpour, greed and corrupt practices and others [18, 19, 20, 21, 22, 23, 24, 25, 26, 27, 28, 29, 30, 31, 32, 33]. Specifically, Oyewande noted that around 50% of the factors leading to building collapse in Nigeria can be linked to faulty design, 40% to fault on construction sites and 10% to product failure [34]. This view was corroborated by Taiwo [35] who observed that the collapse of buildings could be as a result of faults in any or all of the stages in the design, approval, supervision or construction of the building. There is also the finding suggesting that most structural failures and damages in buildings may result from errors in the planning, designing, erecting, and using the buildings rather than statistically prediction of differences in the load bearing capacity of buildings and the load on them [36].

The foregoing suggests that most of the factors associated with building collapse in Nigeria can be linked to human factors. In spite of this, no study known to the authors have examined the causes of building collapse associated with building planning approval process in Nigeria. Since building procurement process involves several stages from pre-design stage to approval and construction stages [37] and errors in each of these stages can contribute to the quality of the final product, it has become imperative to investigate the roles of the different stakeholders in this process so as to identify where gaps and inefficiencies exist that can result to the collapse of buildings under construction or in use. This means that the search for the causes of building collapse must start from the point where building drawings are vetted and approved before construction. Based on this, this study posits that the search for solutions to building collapse and its concomitant effects will be inconclusive if the roles of planning and approval authorities are not properly investigated and adequately understood. This is because town planning and approval are charged with the responsibilities of certifying and approving drawings before buildings are constructed. In the light of the above this research was aimed at investigating the role of town planning and approval authorities in building collapse in Nigeria using Enugu Metropolis as case study. To achieve this aim, this study pursued the following objectives.

1. To identify the staff strength and academic qualification of those involved in town planning and building plan approvals in Enugu Metropolis.
2. To examine the statutory documents required for building plan approval in the study area
3. To identify the aspects of building drawings town planning officers vet before granting approval for the construction of buildings in the study area
4. To determine the effectiveness of site supervision and monitoring as carried out by the town planning officers in the study area.

This study has become necessary to uncover the extent to which physical planning and development authorities have been effective in the discharge of their duties in checking or reducing the growing incidences of building collapse in Nigeria. The research makes contribution to the body of knowledge by revealing the contributory roles of town planning and approval authorities to the growing cases of building collapse in Enugu and its environs. Therefore, the findings of this study are expected to inform policies and practice on the specific aspects of the activities of building planning approval agencies that need strengthening to curb the current growing incidences of building collapse in Nigeria.

## 2 Context of study

**Figure 1:**
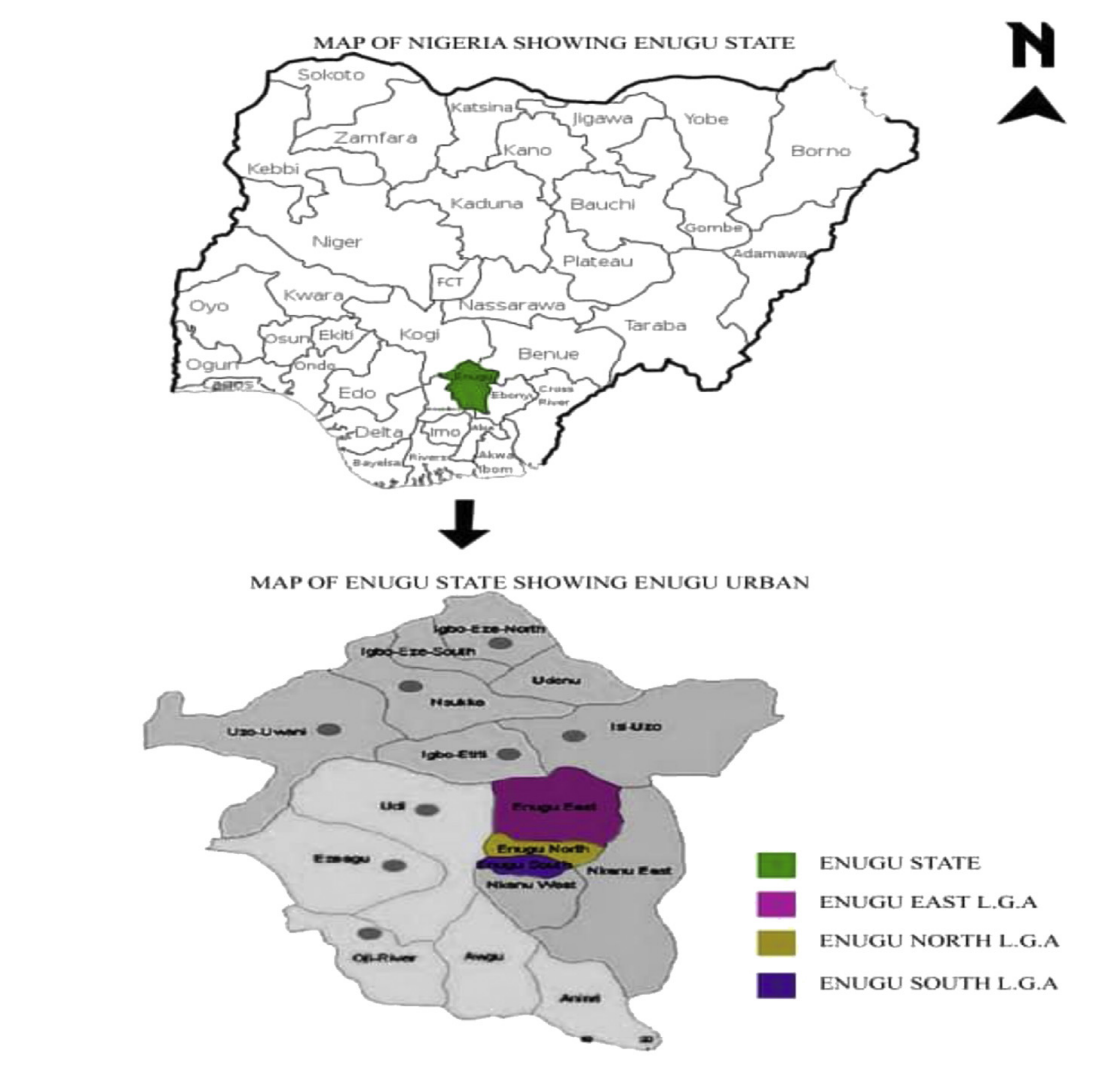
Enugu State indicated in Map of Nigeria and Map of Enugu state showing the study area, (Enugu urban). Source: author (2020).

Enugu is a city in southeast geo-political zone of Nigeria and the administrative capital of Enugu State. The city, which is dominantly populated by people of the Igbo ethnic group, is a medium-size, but fast expanding municipality [38] situated on latitude 6° 27′ 10″ North of the Equator and 7° 30′ 40″ East of the Greenwich Meridian (see Figure 1). Located in Nigeria's tropical rain forest area with a derived savannah and in the basin of the Cross River and the Benue trough, it has a huge deposit of coal.

In the last five decades, Enugu has been experiencing rapid spatial and demographic growth resulting in increased movement of human beings, good and services as well as commercial activities. Since it became the capital of the now defunct Eastern Region after Nigeria's independence in 1960, Enugu has continued to attract both foreign investors and private developers. In addition, the successive territorial adjustments of 1967, 1976 and 1991 resulted to making Enugu the capital of the territory known today as Enugu State. According to the 2006 Nigerian population census figures, this city had a population of 722,664 and ranked 9th most populous city in Nigeria. It covers a total area of 215mi^2^ (556 km^2^) with a population density of 3,400/sq mi (1,300/km^2^).

Enugu is an administrative centre due to the presence of colonial administrative headquarters, state government and three local government council offices. These are believed to have encouraged rural-urban movement and stimulated the demand for residential and commercial office spaces in this city leading to investments in mass housing projects by the government, corporate private sector organizations and individuals. In view of the massive construction activities going on in the city, it has recorded at least six cases of building collapse in most recent times; and thus there a need to investigate the causes and possible remedies to this unfortunate events in this city.

## 3 Review of related literature

### 3.1 Causes, effects and remedies of building collapse in Nigeria: an overview

From the review of the literature, several studies that investigated the root causes and possible remedies of building collapse in Nigeria were identified and summarised in Table 1.

**Table 1:** Causes and remedies of Building Collapse in Nigeria.

Major Causes of building collapse	Major recommendation	Author(s)
Structural defects, poor material, construction defects	Pre-investigation of building materials before incorporation into the building works.	Olusola & Akintayo, (2009)
Poor concrete practices and technology	Improvement of the existing concrete technology. Tests on the quality of cements has to be conducted regularly by the concerned regulatory agencies.	Ede, et al. (2016)
Structural problem due to the presence of smectite in the soils.	Soil characterization must be conducted before building is done. Government should assist people in cases where smectic contents are high.	Una, et al. (2015)
Geotechnical and geological reasons.	Soil characterization must be conducted before building is constructed, especially for high rise buildings.	Oyediran & Famakinwa (2015)
Low quality reinforced steel bars	Continuous random test of building materials is done to determine when specifications are deviated. Tests are to be conducted on the materials to ensure that they are of the required standards.	Adeleke, & Odusote (2013).
Low quality reinforced steel bars	Ensure that quality steel bars are used and sanctions are prescribed for observed corrupt practices as it regards to local production of reinforced steel bars.	Kolawole, & Akanni, (2012)
Lack of pre & post construction investigations, under design, improper supervision, use of substandard material, poor quality funding & construction.	Geotechnical reports must be available before, during and after construction of buildings.	Amadi, et al (2012)
Management, structural defects, construction defects, poor materials, Geophysical, corruption, legal.	Awareness and trainings on the dangers and causes of building collapse should be regularly conducted for stakeholders in the built environment and landlords, property developers and contractors. Safety measures are to be ensured during the building process	Ayedun, et al (2011).
Substandard concrete and concrete materials.	Emphasis on strict adherence to specifications. Closer collaboration among the professionals in the built environment is very key to ensure quality control and assurance.	Oduola (2010)
Flaws in the structure, poor quality materials, construction defects, improper design and dilapidation, foundation issues in buildings on reclaimed lands.	Compressive investigations of the geophysical characterization of building sites are highly recommended.	Oni (2010)
Lack of adequate building survey.	Government and professional buildings should regulate the practice of building survey.	Dahiru, & Okotie, (2010)
Incompetent construction workers, low quality of construction materials.	Recruitment and effective supervision of skilled and competent workers. Quality checks on, and sanitization of the building materials before incorporation into the building works.	Oke & Abiola-Falemu, (2009).

Source; Hilary et al. [39].

**Figure 2:**
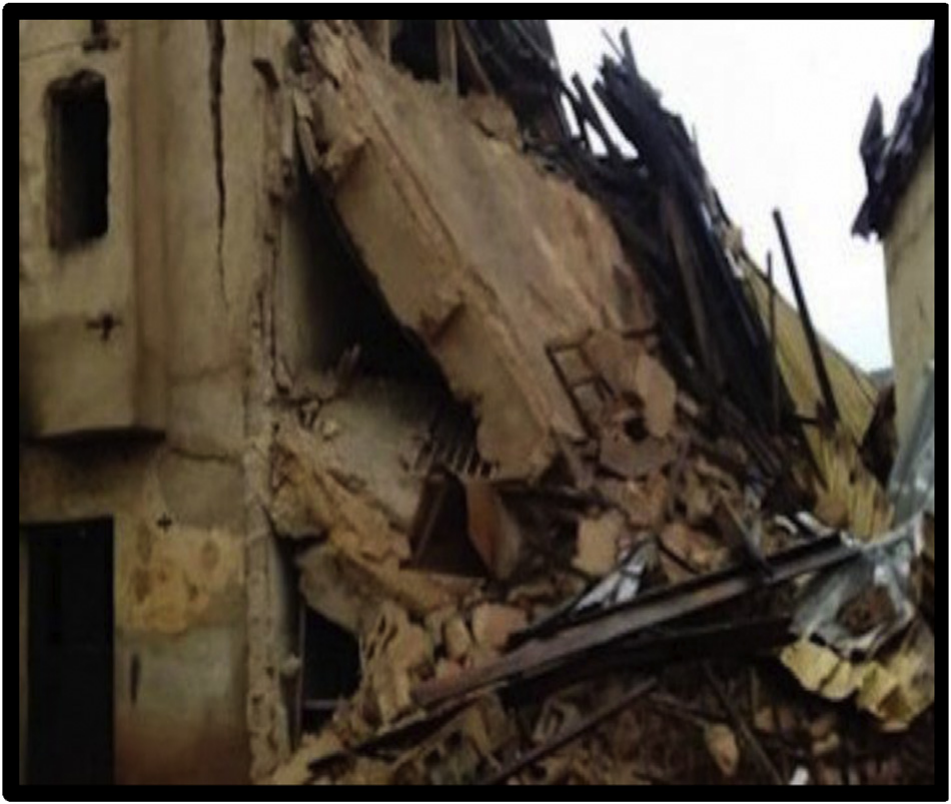
The collapsed building project in under controlled demolition in Lagos State. Source: Authors' (2020).

**Figure 3:**
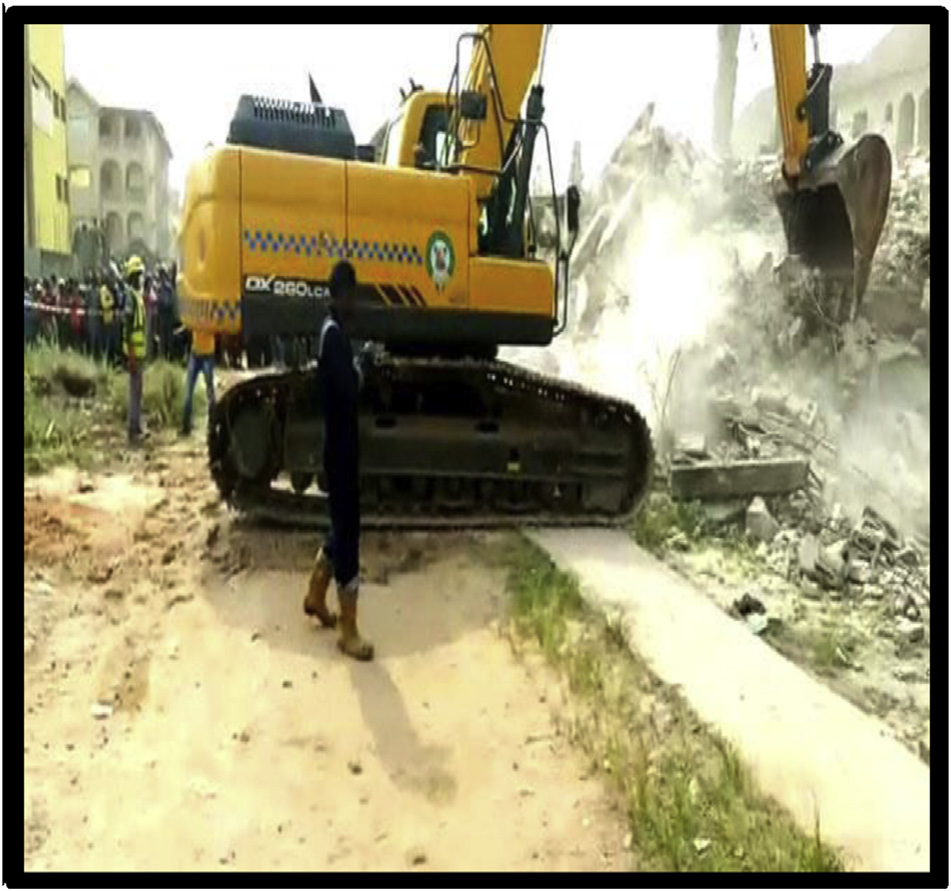
The collapsed building project in under controlled demolition in Lagos State. Source: Authors' (2020).

From Table 1 it can be seen that the existing studies have uncovered various factors linked to the incidences of collapsed buildings in Nigeria. Chief among these are flaws in the design of building structures, poor quality materials, construction defects, improper design and dilapidation, foundation issues in buildings on reclaimed lands. Others are improper supervision, poor quality reinforcement bars, geotechnical and geological reasons. In addition, evidence in the literature also indicate that poor staffing, civil service protocols, lack of experience, faulty methods, sentiments, cowardliness, acceptance poor standard materials and works, corrupt practices and lack of enforcement are the key challenges confronting the efficiency and effectiveness of physical planning and building control operations across Nigeria [40]. In fact, Folagbade and Badejo alluded that the common factors that can lead to building collapse in Nigeria include lack of adequate supervision, ineffective enforcement of building regulations by the Town Planning Authorities and poor maintenance culture [41, 42]. For examples, the findings of the Lagos State Emergency Management Agency on the collapse of a 3 storey residential building under construction at Ugochukwu Omeja Close off Alasepe Street/Community Road off Ago Palace Way, Okota Lagos State Shown in Figures 2 and 3 that collapsed on 17^th^ January, 2020 (see Figure) revealed that the primary cause of the collapse was poor supervisory role of Planning Authority, while the secondary cause of the collapse was linked to poor workmanship and the use of substandard building materials.

**Figure 4:**
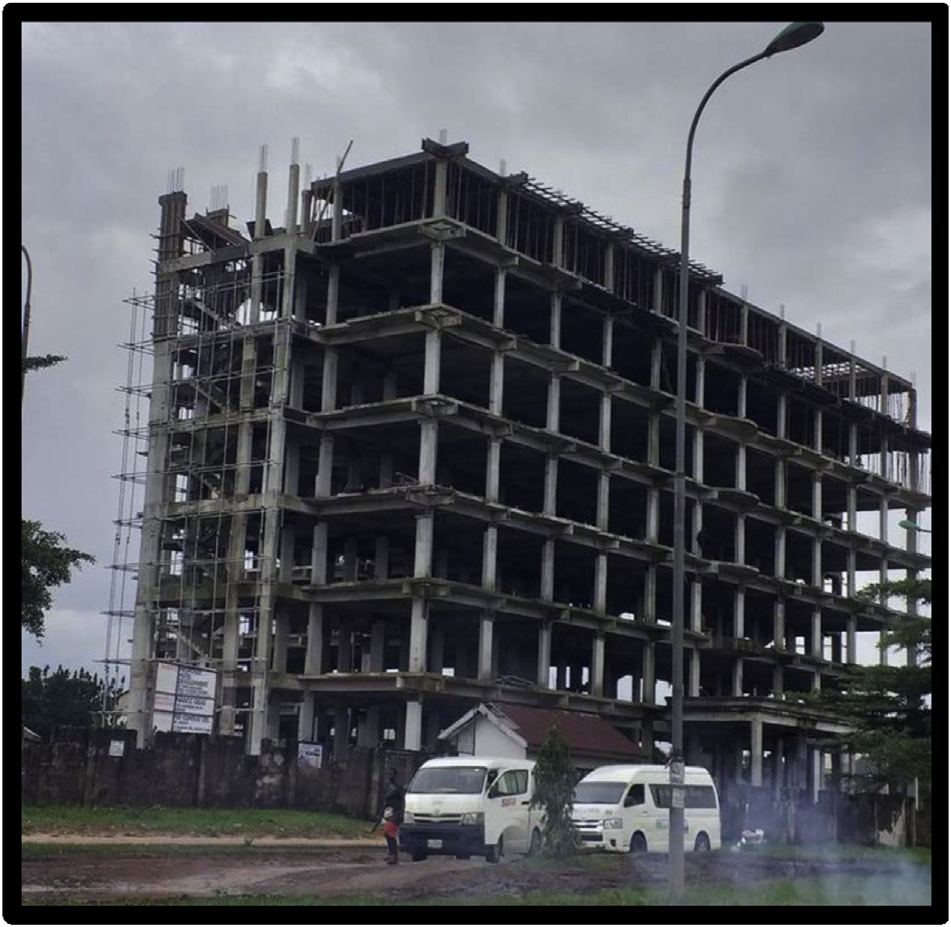
8-storey hotel building before collapse in Owerri Metropolis Imo State. Source: Author (2020).

**Figure 5:**
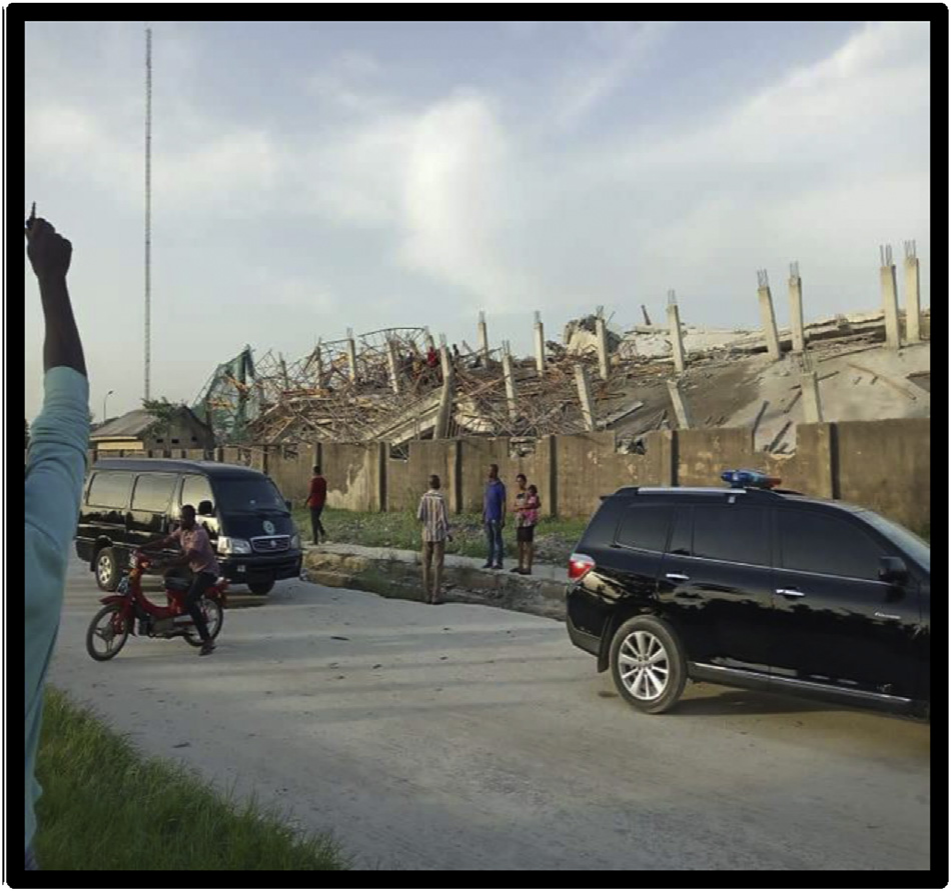
8-storey hotel building after collapse in Owerri Metropolis Imo State. Source: Author (2020).

Similarly, the preliminary reports of the panel of investigation comprising the Owerri Capital Development Authority (OCDA) and the Nigerian Institute of Architects (NIA) on the collapse of a 8-storey hotel building under construction located along Musa Yar' Adua Drive, Owerri, Imo State shown in Figures 4 and 5 that collapsed on 30^th^ April, 2020 resulting in 40 fatalities, identified the primary cause of the collapse as non-adherence to construction drawings, poor supervisory role of planning authority and secondary cause of the collapse to be the use of substandard building material and structural failure.

Furthermore, the study of Adeyemi [19] revealed that non-enforcement of building codes, non-professionals, unqualified builders and poor supervision from government agency among many other factors he listed contributes to building collapse in Nigeria. The author also revealed that bad design is a cause of building collapse in Nigeria. These submissions raise a very pertinent question regarding the effectiveness and efficiency of planning approval authorities in Nigeria. Obviously, one can infer from here that building drawings are either not properly assessed and scrutinized for errors or are being assessed by non or incompetent professional.

Regarding the remedies, the authors cited in Table 1 suggest that among several measures, awareness and trainings on the dangers and causes of building collapse, implementation of safety measures during the building process, recruitment and effective supervision of skilled and competent workers, quality checks on building materials, and regulation of the practice of building survey should be instituted in the construction industry. From another perspective, the report of the Former Honorary General Secretary of the Nigerian Institute of Architects, on building collapse in Lagos State in 2013 revealed that although a summary trial of infringers has been provided for in the law, but due to cultural, political administrative factors and other interventions, one cannot find any evidence suggesting that persons have been sanctioned or prosecuted by any legal entity for contributing to building collapse in Nigeria [43]. This suggests that the enforcement of the existing laws regarding the roles of individuals and organisation in building collapse in Nigeria is very weak. Hence, it has been suggested that appropriate sanction of persons and or organisations who have been found to contribute to building collapse will serve as a deterrent to others as no amount of compensation will be adequate for fatalities resulting from building collapse [44]. It was based on this premise that stakeholders in the Nigerian construction industry have stressed that the issue of building collapse will persist in this country until prosecutions are brought against those who contravene building codes, regulations and bye-laws. In line with this, the Council for the Regulation of Engineering in Nigeria (COREN) has proposed capital punishment for proprietors of faulty structures [45].

### 3.2 Legislative and human resource composition of the Nigerian building industry

The building industry is among the most complex and multifaceted sector of the economy of any nation. The industry employs a wide range of stakeholders [46] including foreign and local professionals, materials, labour and equipment that co-habit, interact and function in order to realize building projects. It is made up of design professionals (architects, engineers), cost consultants (quantity surveyors) construction team (builders and contractors), town planners and land surveyors as well as support group (public administrator, developers, financial institutions). For this reason, a town planning and approval authority is constituted to oversee the approval of all construction project within a particular geographical location. There are several enforceable pieces of legislation, standards and regulations that govern the operations of the building industry, which have been designed to facilitate safe and conducive work environment required of the delivery of structurally sound buildings. During construction stage of buildings, permits and approvals or certifications are usually required as a prerequisite for moving to the next stage of the work. Such permits or approvals are legal requirement and as such special care and attention must be given to meet the established requirements failure of which may result in liability on the part of those involved in the construction process.

The Nigerian Urban and Regional Planning (NURP) Law (decree 88 of 1992) and National Urban Development (2006) are the main legal/policy instruments that outline the roles of the federal, state and local governments in urban and regional planning with hierarchy of plans and bottom-top feedback. The Constitution of the Federal Republic of Nigeria (CFRN) 1999 puts the standards for building/construction projects under in the concurrent legislative list; meaning that building standards are set by all tiers of government: federal and state and local governments, but where there is a conflict the federal standards override all the standards. Resulting from this, there are building codes, regulations and bye-laws at federal, state and local government levels in Nigeria Although building standards in Nigeria have evolved over the years from customs, practices and existing materials, best practices in building design, materials use, safety requirements, labour, professions, monitoring and management are continually being modified in line with the dynamics the various stages of building works [47].

Arising from the various pieces of legislation guide building standards in Nigeria are statutory regulations, which set minimum standards that must be complied with and non-statutory guidelines, such as the national building code and code of conducts or ethics for professionals in the industry. All these complement each other to ensure the integrity of building projects, workers welfare and safety, public and environmental health practices. Some of the pieces of legislation applicable to the industry include the Standard Organization of Nigeria Act (SON Act), the Land Use Act of 1978 as amended in 2004, the Factories Act of 2004, National Building Code 2006 (NBC), Labour Act 2004, Industrial Training Fund Act 2011 and several State laws. Although these pieces of legislation have been put in place to ensure adequate human and environmental protection, their relevance in curbing the rising cases of building collapse have not been adequately felt across the country as cases of building collapse are on the increase unabated.

### 3.3 Statutory documentary requirements for building plan approval in Nigeria

In Nigeria, physical planning and development activities are regulated by the Nigerian Urban and Regional Planning Decree (NURP) No 88 of 1992, which allows for decentralization of physical planning and control activities through the establishment of institutions at three tiers of government. At the federal level, is the Urban and Regional Planning Commission, at the state level is the Urban Development Boards and at the local government levels, the Local Planning Authorities. These agencies are not only to implement the provisions of the NURP law 1992, but also the National Physical Development Plan (NUDP) which was first launched in 1997 and other policies, programmes and activities required to advance town and city planning and improve the lives of the people [48]. Subsequently, Section 7.4 of the Nigerian National Building Code 2006 elaborated on statutory documentary requirements for building plan approval in Nigeria and conditions that shall be satisfied with regard to regulation of physical planning as required by appropriate planning authorities in their areas of jurisdiction. The physical planning requirements are stated as thus [49]:

(i) Evidence of Site Acquisition, Survey Plan and Title Deed: Building plots shall have evidence of Right of Land ownership, Site Survey Plan and Deed of Assignments - Right of Occupancy, sworn affidavit from court on facts or Certificate of Occupancy.
(ii) Building Site Location Plan: This document is required for ease of site identification in unplanned neighbourhood; in most cases this is contained in the architectural working drawing.
(iii) Site Analysis Report and Plan (SARP): This document prepared by a registered town planner to ensure conformity of the proposed development in the neighbourhoods. All proposed building development are required to conform with existing Urban Design Plan and zoning regulation.
(iv) Designs Drawings: Depending on the geographical location, a minimum of 3 set of building plan made up of architectural working drawings, structural engineering drawing, mechanical and electrical engineering designs are required for obtaining development Permit.
(v) Environmental Impact Assessment (EIA): This document is required for proposed non-residential development to establish the impact of proposed development on the immediate environment in accordance with the EIA Law where applicable.
(vi) Tax clearance: This is to verify if the citizen is fulfilling their civic duties, depending on the area of proposed development a minimum of 3 years' tax clearance is required most times if the land is located in a government layout or urban centres.
(vii) Official fees for town planning development: A development fee is to be paid to the local authority. However, it varies based on building type of development, building height, density, location of development, and its environmental impact within the neighbourhood.
(viii) Other special consideration: In large development projects, Certification from appropriate regulatory body or statutory agency on the intended use of special material, equipment or licenced personnel is a mandatory requirement.

### 3.4 Building drawings, supervision and monitoring of construction sites and building collapse

The important aspects of building drawings, supervision and monitoring that are directly linked to preventing building collapse as contained in the existing legislation extracted from the Nigerian National Building Codes 2006 [49] that needs to be checked in building plan drawing and verified on site during construction are described in the next paragraphs.

#### 3.4.1 Requirements of architectural and structural drawings

Architecture building plans are to be drawn on suitable scales to ensure the readability and clarity of the drawings with all relevant details, sections and callout included in order not to leave room for assumptions during site work. The floor plans of buildings must show the use and dimensions of each space, door, door handle and window schedules, sections and gridlines. The architectural drawings must also be checked for the type of use and occupancy to conform with the location, building type and prevailing zoning ordinance. To maintain environment justice, building design site plan must be within for property line, respect easement standards, the right of way and not encroach into natural reserves or obstruct water ways. In addition, site location plan showing access in and around the building, setbacks to the property lines, parking space, location and distances to other buildings, details of vehicular turn radius are required. Minimum requirements for room space, size and building height also in relation to other existing building must be checked for adherence to the stipulated standards.

Structural drawings are to conform to the British Standard being used in the Nigerian construction sector. In building design, several categories of loads are considered and the structural elements, including beams, columns and slabs designed to support these loads. The location and sizes of both the architectural and structural elements in the building are shown in the architectural and structural drawings, respectively. Similarly, material specifications and finishing schedules are also included in the drawings and these are usually expected to be within the approached standard for the different building type and site conditions such as bearing capacity and topography. The National Building Codes 2006 also stipulates that the structural framework should comprise elements such as columns and the girders, beams, slab, trusses and spandrels having direct connections to the columns and all other members, which are important in ensuring the structural stability of buildings as a whole and must be thoroughly scrutinize and vetted in the drawings.

The structural calculation sheets, bending schedules, grades of cement and mixtures should be stated in the drawings and be verified, while material and reinforcement bar test are to be carried out and certified in all construction sites. For design load, the structural drawings are to be carefully assessed from substructure to roof design. Foundation design is to be checked in conformity with the results of the soil test to ensure that the soil bearing capacity within the allowable limit of the estimated load on the proposed building. Also to be checked for the total design load in the drawings to ensure it can support safely all loads (dead, live and wind) to be imposed on the structure without exceeding the allowable stresses for the material used in the construction of structural elements of the building. For buildings undergoing alteration or change of use, it is recommended that structural analysis or integrity test be carried out to determine adequacy of all structural systems for the proposed alteration, addition or change of use. This is to ensure that building the is capable of supporting the new load resulting from the alteration or change of use.

#### 3.4.2 Requirements of services drawings

The National Building Codes 2006 also make its mandatory that services drawings of buildings of over two stories in height be prepared to show locations of electrical, mechanical, plumbing and communications conduits, pipes and systems, as well as the materials and methods for ensuring structural integrity, fire resistance rating and fire-stopping. Also to be verified in the services drawings and on site are all electrical calculations, power loading and specifications to comply with standard building practise and intended electric design load for the building type. For mechanical drawings, the code recommends that this should show the location of duct layouts and sizes, fire/smoke dampers, location of mechanical equipment/installations (pumps, piping, sprinklers, rising mains, fire suppression systems, etc. on the roof, ground or wall, while cross sections of roof showing mechanical equipment/installations and detailed schedule of equipment and sizes, schematic of air distribution line diagram for the proposed system, specification for air volume capacity of fans and other air handling equipment and calculations should also be incorporating the proposed system. In the same vein, the electrical drawings should show clearly the locations of main electrical service and all sub-panels, one line diagrams of all major wiring, location of all outlets, switches and light fixtures, fire alarm and detection system, emergency lighting, exit signs, duct fire detectors for HVAC and detailed schedule of equipment and ratings/calculations.

Regarding fire safety requirements, provisions for fire protection equipment are required in all building plans or structures and these are to be checked for adequacy in all drawing and verified on site during the construction stage to ensure they all conform to standards. In addition, site location plan showing the position of fire hydrants, underground utilities, access to fire engines/equipment is required as well as compartmentation. The details for rated building elements such as walls, floors, ceiling assemblies, columns, girders and beams are also needed, while fire resistance rating of load bearing beams and columns, foundation, floors, roof plans and related structural details, minimum design loads and materials standard references for concrete, steel, wood and others should also be provided and checked for accuracy and consistency. For the purpose of evacuation during fire outbreak the Nigerian National Building Codes 2006 [49] also requires that life safety plan be produced to show the use for each space with occupant loads based on occupant load factors, exiting paths for exit of occupants and capacity of exit doors or exit stairways, travel distance; smoke barriers, fire resistance of rated partitions and firewalls with appropriate legends and signals. It also stipulates that the position of breeching inlet(s), dry/wet riser outlets and hose reels, whichever is applicable, shall be indicated in red colour on floor plan. All these components are meant to be scrutinised and vetted by officials in the Town Planning and Building Approval offices before building permits are issued to developers.

## 4 Research methodology

**Figure 6:**
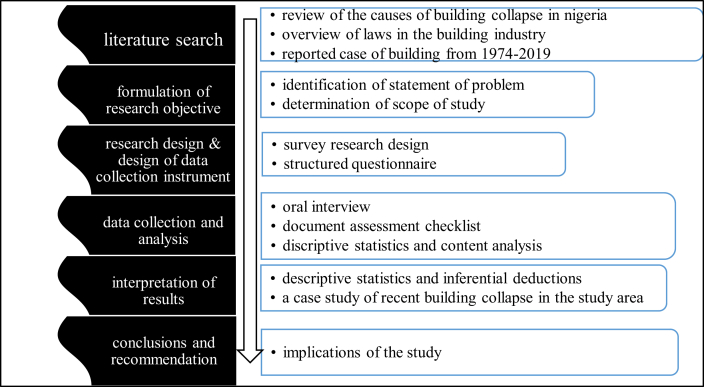
Research methodology flow chart. Source: Authors' conception.

This research followed a survey research design. The graphical illustration of the research methodology flow chart is shown in Figure 6. From Figure 6, it is evident that research process can be broken into six main stages starting with literature search to drawing conclusion and making recommendations. The research involved gathering both primary and secondary data from different sources. The secondary data were obtained from the review of published research works and the National Building Codes 2006. The primary data were sourced from oral interviews, administration of questionnaire and non-participant observation. The research population for the questionnaire survey comprised works in the Town Planning and Approval offices in the three Local Government Areas (LGAs) (Enugu North, Enugu East and Enugu South) that make up Enugu Metropolis. The questionnaire instrument designed by the authors had five-items that required the participants to provide information regarding staffing of the approval offices, the requirement and document for building plan approval, their duties and other responsibility the authorities. Other data were derived from examination of the planning office document assessment checklist. The reason for selecting this research instrument is because it allowed the researcher to specify a measurement procedure in detail in order to define the quantity of a variable. In the administration of the questionnaire, all the 86 staff members identified in the three LGAs Town Planning and Approval Authorities were sampled. The distribution of the staff according to their LGAs is shown in Table 2.

**Table 2:** Statistics of workers in Enugu metropolis town planning and approval offices.

Staff qualification	Enugu South n (%)	Enugu East n (%)	Enugu North n (%)	Total number of staff n (%)
Number of Town planners	3 (37.5)	2 (25.0)	3 (37.5)	8 (100.0)
Number of Administrative staff	18 (23.1)	39 (50.0)	21 (26.9)	78 (100.0)
Number of Architects	0 (0.0)	0 (0.0)	0 (0.0)	0 (0.0)
Number of Structural Engineers	0 (0.0)	0 (0.0)	0 (0.0)	0 (0.0)
Total number of staff	21 (24.4)	41 (47.7)	24 (27.9)	86 (100.0)

For the oral interviews, a reconnaissance survey was used to 30 contractors in ongoing construction projects within Enugu Metropolis, comprising of 10 sites from each of the three Local Government Areas to validate the response from the Planning Authorities. This is because of the high level of secrecy and reluctance by contractors to give out information due to fear of the unknown. The interviews with 30 contractors was based on random sampling technique and were centred on the frequency of site visit by officials of the Town Planning and Approval Authorities to monitor the level of compliance of the construction work with the approved plans and conduct the necessary material tests. The interviews were recorded by taking notes by the researchers. In addition, a case study of the most recent building that collapsed in Enugu metropolis was done. The site for the most recent case of building collapse in the study area was identified and visited. The visit assisted the researchers to have on the spot observation of this case and document the available evidence on the site. The key data collection instrument used is photographic materials.

The data obtained from the fieldwork were collated and analysed using descriptive statistics and content analysis. The results are presented using tables, texts and photographs for a better understanding.

## 5 Results

### 5.1 Staff strength

The research was conducted in Enugu metropolis which include Enugu North, Enugu East and Enugu South is presented below. The result in Table 2 show the statistics of workers in Town Planning Approval Authorities in Enugu metropolis.

The results (Table 2) reveal that the available workforce is 86 staff of which only 8 Town Planners are the only professional who do the actual vetting and approval of drawing in the town planning and approval offices. This represents a very small percentage as compared to other administrative staff including health workers that sum up to make the highest percentage of 78 staff. The 3 Local Government Town Planning Authority visited have No Architect, No Structural Engineer, No Electrical engineer, No Mechanical engineer and No Soil Test Engineer in their workforce.

### 5.2 Document required for building plan approval in Enugu urban

From the survey conducted, below are the requirement for building approval in Enugu metropolis.

1) 4 set of building plan (architectural, mechanical, structural and electrical)
2) One survey plan of the site.
3) Evidence of ownership (C of O, deeds of assignment, deed of need)
4) 3-year tax clearance. (if the land is located in a government layout, you obtain clearance from zonal officer in the ministry of lands)
5) Affidavit from court on facts.

When proposed building Plan is submitted to the town planning office; they assess the plan and give a bill for fees to be paid. It includes;

i. Official fees for town planning
ii. Capital territory fees
iii. Fees for site analysis report and plan (SARP)

### 5.3 Aspects of building drawings vetted before granting development approval

After survey and interview with town planners in the various approval offices, their standard and criteria for approving drawing assessment are listed below.

1) They make sure that the rules for setbacks are followed. That is 3m for sides and back of all buildings with 4.5m for the front for residential development and 6m for commercial development.
2) They make sure building project go along with the existing scheme. Whether the area is just for bungalow or maisonette.
3) Check the ventilation standard whether it is cross ventilated.
4) Check the comfortability of the room in terms of the room length and width of not less than 3m and the toilet provision.
5) Height of the building in relation to the density of the area.
6) Zoning and the prevailing land use ordinance of the proposed site.
7) Check the property line to avoid encroachment into any neighbouring site
8) Check the soak away and septic tank detail if it can meet the need of the development.

The whole vetting and approval process takes no longer than 3 months.

### 5.4 Effectiveness of site supervision and monitoring

Table 3 shows the checklist for construction site visit and frequency of visit by Local Government Town Planning Authority in Enugu urban. The survey gathered that staff who undertake this assignment are administrative workers. For the three local government under study the checklist for inspection is the same likewise the fact that there is no scheduled dates or period for site visit (supervision) and monitoring, but done according to their whims and caprices.

**Table 3:** Frequency of site visits and check list of local government town planning authority in Enugu metropolis.

	Enugu south	Enugu east	Enugu north	Remark
Criteria of inspection	• Setbacks. • Septic tank and ventilation standard. • Check if as built is as approved. • Check materials on site.	• Check if as built is as approved. • Zoning regulation. • Setbacks. • Check materials on site.	• Check if as built is as approved. • Check materials on site. • Building line and setbacks.	Uniform check list
Frequency of site visit	Often as possible but no regular routine for site visits	As much as possible	Site visits is at will but response faster to conflict sites	No scheduled time for site visit
Staff who visit site	Administrative staff	Administrative staff	Administrative staff	Same Staff

Table 4 contains data of reconnaissance survey collected by means of interview from developers and site contractors to verify the frequency and effectiveness of local government town planning authority in discharging their duties of effective monitoring, supervision and implementation of building codes and regulation of ongoing construction project within their jurisdiction.

**Table 4:** Survey of Developers in ongoing construction projects in Enugu.

Frequency of site visit	Enugu south LGA. building construction sites	Enugu East LGA. building construction sites	Enugu North LGA. building construction sites	Total number of site visited
Daily site visit	0 (0.0)	0 (0.0)	0 (0.0)	0 (0.0)
Weekly site visit	1 (33.3)	0 (0.0)	2 (66.7)	3 (100.0)
Monthly visit site	2 (28.6)	1 (14.3)	4 (57.1)	7 (100.0)
Never visited	7 (35.0)	9 (45.0)	4 (20.0)	20 (100.0)
Total	**10 (33.3)**	**10(33.3)**	**10(33.3)**	**30(100.0)**

The results in Table 4 reveal that three out of 30 sites reported weekly visits, 7 reported monthly visit and 20 reported no visit at all after paying approval, sundry, and material testing fees.

**Figure 7:**
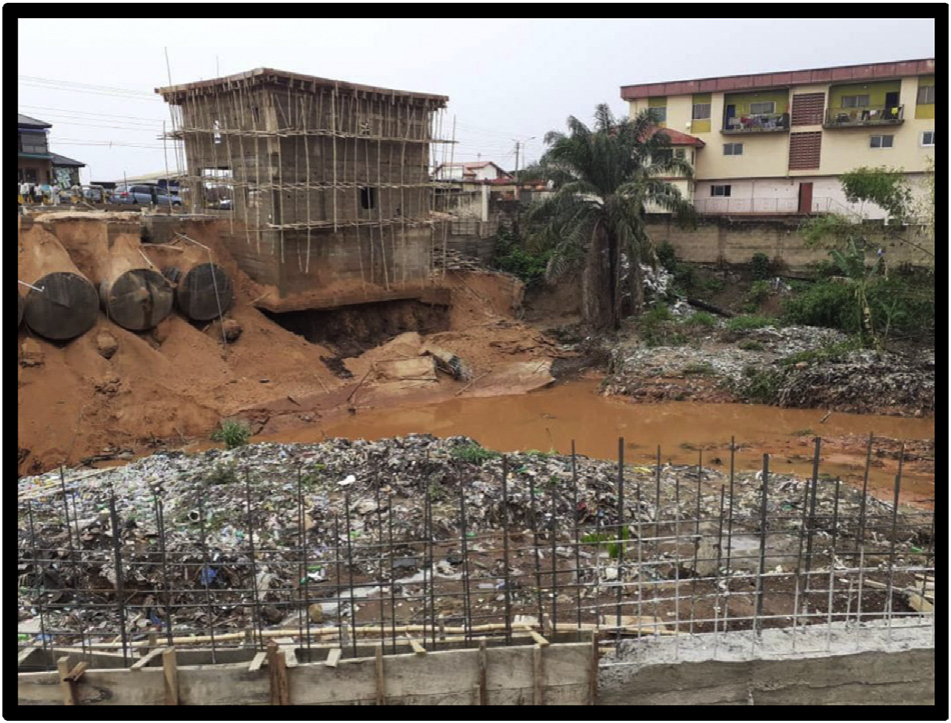
The building project on water ways along O'connor Street, Enugu. Source: Authors' fieldwork (2020).

**Figure 8:**
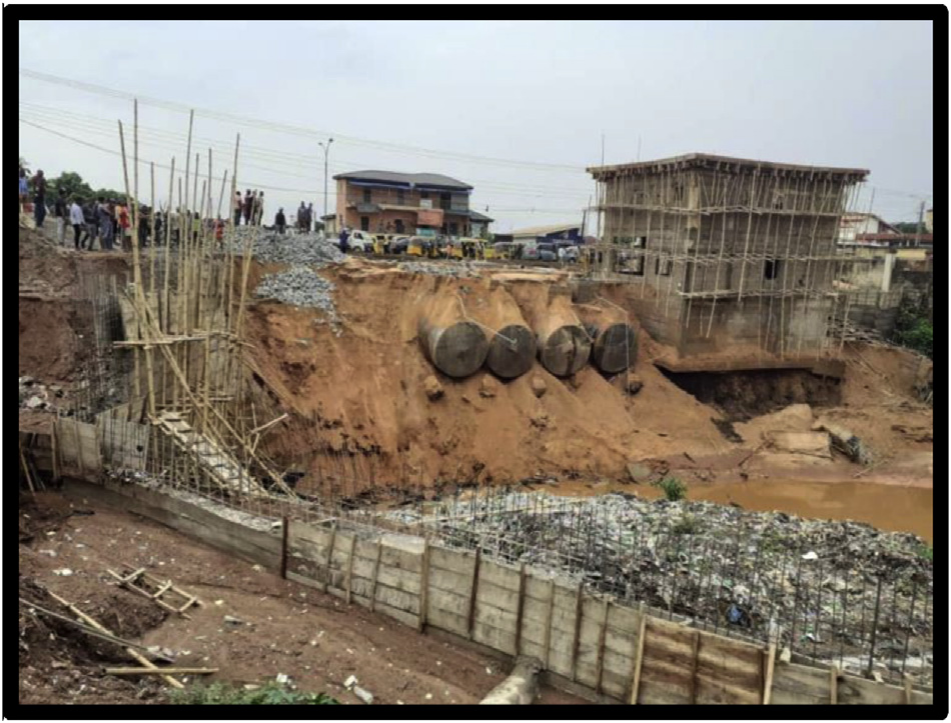
The building project on water ways along O'connor Street, Enugu. Source: Authors' fieldwork (2020).

**Figure 9:**
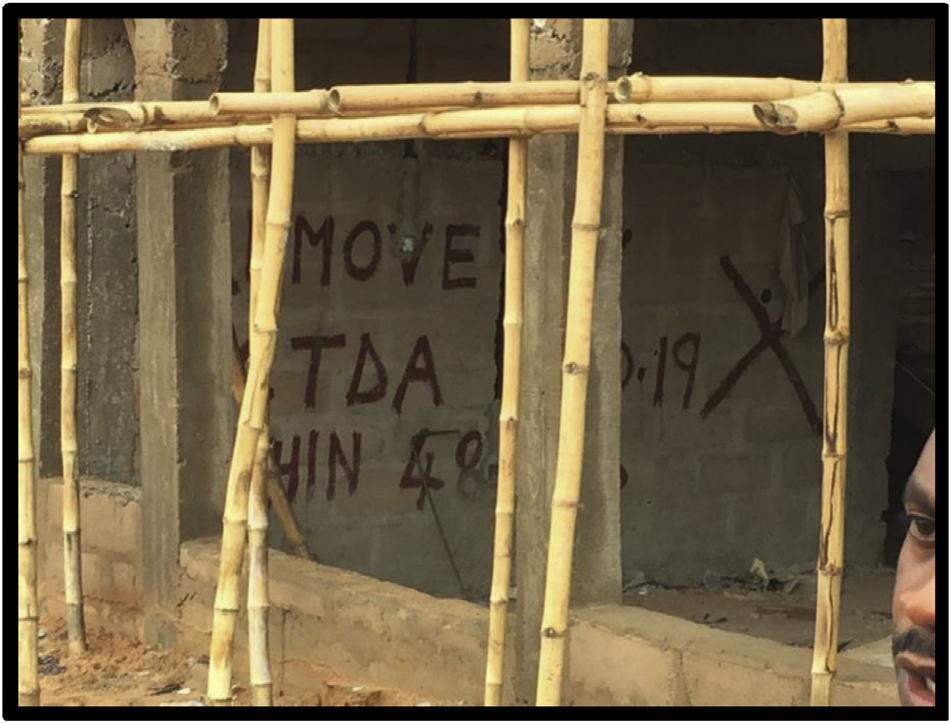
The building project mark of stop work order by ECDA along O'connor Street, Enugu. Source: Authors' fieldwork (2020).

**Figure 10:**
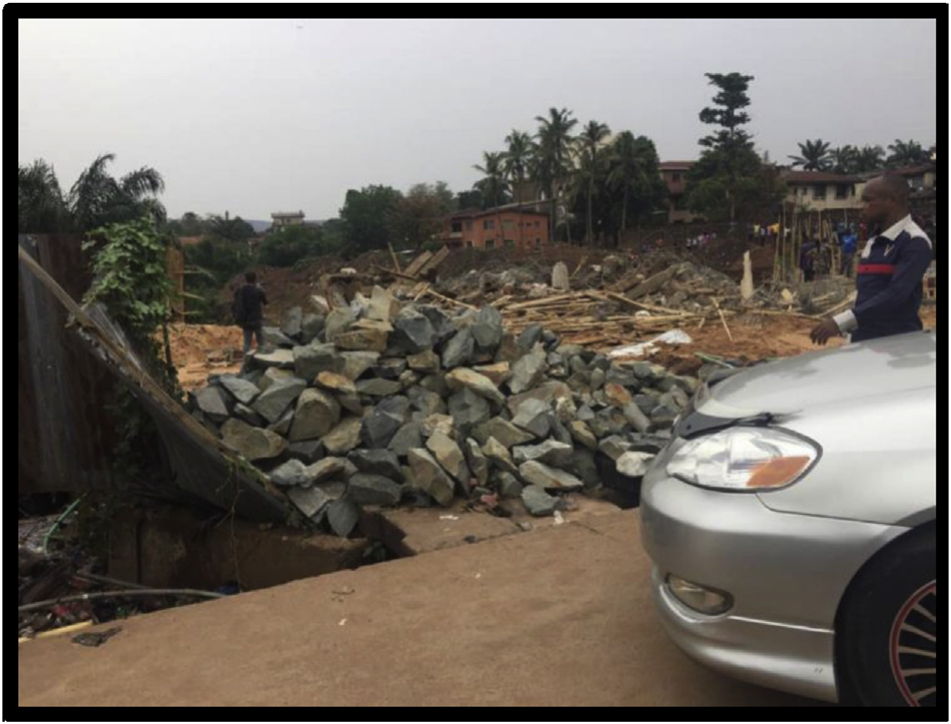
The debris of collapsed site along O'connor Street, Enugu. Source: Authors' fieldwork (2020).

Figures 7, 8, 9, and 10 show the pictures of the case study of the gas station under construction along O'Connor Street, Enugu Metropolis that collapsed in April 2020. According to the investigative panel comprising the Enugu Capital Development Authority (ECDA) and the Nigerian Institute of Architects (NIA) revealed that although no fatalities was recorded, the main causes of the collapse of the building were the lack of building approval (no development permit was issued to the developer), poor supervisory role of planning authority and corruption. The secondary causes of the collapse were linked to heavy rainfall and location of the building along storm water channel.

## 6 Discussion

From the findings of this research, we have identified four key issues related to the four research objective stated for this study for further discussion here. The first is the findings on the human resource composition (staff strength) of the Town Planning Authorities in the study area. From the results of the survey (see Table 2), it is evident that in the Town Planning and Approval Authorities investigated, there is a lack of adequate and proper staffing. In fact, it was observed that of the 86 members of staff identified in the Town Planning Offices in the study area, only 8 staff, representing just about 9.3% are saddled with the responsibility of assessing, scrutinizing, evaluating and approving building document and construction drawings. It was also found out that none of the 8 professionals vetting architectural drawings and granting approvals was found be to architect, and thus, they are considered to have limited knowledge of architectural designs and buildings. Similarly, the planning offices were also found to have no structural, electrical, mechanical and soil engineers. In view of these findings, one becomes curious of who vets engineering designs as the existing staff in these offices have little or no formal training in building designs may not have the capacity to detect errors or omissions in working drawings submitted by developers for vetting and approval.

Notably, conception, analysis and execution and other unforeseen errors may occur even when qualified personnel has been engaged in the process of designing and erecting buildings and when appropriate methods and necessary quality assurance and control measure have been employed, the peer review process in the vetting and approval of drawings in the Town Planning Authority can help to detect some of these errors of omission. However, in a situation where there is absence of qualified personnel to carry out the vetting assignment, this aspect of quality control in the building planning approval process will definitely suffer. This is indeed a missing link in the process of building plan approvals by government agencies in the study area, which is capable of contributing to the increasing cases of building collapse. This specific finding on the inadequacies of government agencies in Nigeria is in line with the research of Fagbenle and Oluwunmi, who blamed the growing number of collapsed buildings on poor compliance with approval of building plans prior to construction, dysfunctional monitoring protocols employed by the relevant government agencies and poor awareness of the existing building regulations and bye-laws by clients/contractors [50]. It is also consistent with that study by Oyedele et al indicating that the rising cases of collapsed buildings in Nigeria can be linked to inadequate number of supervisors from government ministries, inadequate provision of logistics required for effective monitoring of construction works and corrupt practices [16]. This finding also seems to be in line with the result of Asante and Sasu who reported that those responsible for inspecting buildings in Kumasi, Ghana have thus far failed to control the rate of building collapse in that country due to several challenges, including poor logistics, inadequate manpower and excessive political interference [2].

The second relates to the finding on the documents required for granting building plan approval in Enugu urban. This study found that results of soil test of site for proposed development is not a requirement for approving building plan. Since loads on buildings are transmitted to the soil through the foundations and the soil bearing capacity determines the type of foundation of the building and the magnitude of the load soil can support, lack of attention to the type of soil and its load bearing capacity by Town Planning and Approval office in the study area is considered a major issue, especially when multi-story buildings are involved. Although results of soil investigation are supposed to provide key data for the structural engineer to design the foundations of the building, the documents showing the result are supposed to be part of the construction documents submitted for vetting in the Town Planning and Building Approval Office. This is important in verifying the extent to which the design of the foundation of the proposed building is in line with the recommendations of the soil engineer. The non-attention to soil test by Town Planning and Building Plan approval authority in the study area can be linked to the absence of Geotechnical Engineer in the approval offices to handle this aspect in the study area. This suggest that building plans approval agencies in the study area are actually neglecting a vital component of building requirement, which deals with the type of soil condition and it bearing capacity. It is known that if the soil bearing capacity is below the design load of the building and the right choice of foundation design was not made, the probability of the building to fail would be higher. This finding is in line with the submission by Oloyede suggesting that cases of building collapse are on the increase in Nigeria due to negligence in soil investigation, the determination of design loads, excess wind and earthquake loads, bad terrain, the use of poor quality building materials, inadequate monitoring by the relevant government agencies and poor workmanship [21]. This was corroborated by Dimuna who also noted that the absence of soil testing prior to commencement of construction work, lack of coordination between professional bodies and town planning authorities, and poor enforcement of the existing laws regulating physical development were among the leading causes of building collapse in Nigeria [51].

The third issue relates to the aspects of building drawings the building permit authorities vet for granting building approvals in the study area. Based on the data presented in section 5.3 as a criterion for development control standard for local town planning authorities in Enugu it is evident that these criteria fall short of the standards provided in the National Building Code 2006 as elucidated in the review of literature. It is evident from this study that the assessment criteria seem to be skewed toward the architectural design requirements alone with less emphasis on the structural components. This suggests that the structural drawings which shows the size and location of structural element in buildings are not properly scrutinized to for example to verify how the loads were estimated, calculation sheet and the different sizes and grades of reinforcement specified in the drawings. It is also evident from Section 3.4 that material specifications, electrical and mechanical drawings all not given priority attention by the approval agency in Enugu metropolis. Arguably, the inability of town planning authorities to ascertain that architectural and engineering designs conform to the established standards prior to granting approvals and building permits can contribute to building collapse. According to Omenihu, although Nigeria has a relatively good environment and weather compared to other countries of the World, she is confronted with increasing cases of building collapse due to human factors [17]. Similarly, Ayinuola and Olalusi have also linked the increasing cases of building collapse in Nigeria to clients, contractors, and professionals in the building industry as well as town planners employed in local authorities [52]. This goes to confirm that human actions and inactions play significant role in the rate of building collapse in Nigeria.

Fourth is the findings on site supervision and monitoring by the Town Planning Authorities in the study area. From the results of the survey (see Table 3), it is evident that the uniform check list for building construction project supervision and monitoring within the three local government (study area) fall short of approved standard in the existing building Code. The frequency of construction sites visit for example was also not specified; suggesting that site monitoring and supervision are done arbitrary depending on when the officers deemed fit to undertake the assignment. Their regular/attendance to site during construction is relatively low or poor (see Table 4). In fact, twenty out of thirty site contractor interviewed reported that no staff of the Local Town Planning Authorities visited their sites for inspection and monitoring after their levels of compliance with approval and material testing. This situation can also be linked to the inadequate manpower in the agencies investigated. Furthermore, the study also found out that the few government employees charged with the tasks of vetting and granting building approvals often engage in corrupt practices such as bribery and ignoring of infractions, fraudulent granting of approvals and certifying unsafe buildings. This explains why the developer of the gas station (Figures 7, 8, 9, and 10) they claimed was not granted development approval would commence development within the heart of the town for many weeks unnoticed. This indicates that either the officers are not up and doing in their responsibilities or they collude with developers to circumvent the laid down procedure for building plans approval in the study area.

Since, physical planning activities in Nigeria is regulated by the Nigerian Urban and Regional Planning Decree (NURP) No 88 of 1992, and are monitored by government agencies at the three tier of government, the findings of the current study presented here may not be different from what are obtainable in many towns and cities in Nigeria because the Town Planning Authorities in Nigeria have the same structure and modus operandi. This assertion is based on the evidences of building collapse in Lagos, Owerri and Enugu cited in this paper, which linked these event to among other causes non-adherence to construction drawings and poor supervisory role of the local Planning Authorities. Historical data indicate that most of the reported cases of building collapse in Nigeria were buildings under construction and less of those in use [36]. This has continued to be the case as a majority of recent building collapse reported and cited in the paper are buildings under construction, suggesting that there are a lot of construction malpractices going on in this country.

Generally speaking, the results presented here seem to provide support to the evidence in the literature [39, 53] showing that in the 221 cases of building collapses reported in Nigeria between 1974 and 2019, structural defects/substandard materials, faulty design/carelessness, poor supervision/non-adherence to design standard are the leading cause of most of these cases. This is an indication that the site supervision and monitoring roles of the town planning and approval offices are not being effectively executed; and if this trend continues, it is predicted that more cases of building collapse will be recorded in future. This finding seems to be consistent with the submission of previous authors noting that almost all the cases of building collapse recorded in Nigeria are linked on either government agency whose duties are to ensure compliance or the developers for failure to comply with building regulation or consultant's instruction [34]. Resulting from these lapses in monitoring role, there is the increasing use of poor quality and sub-standard construction materials and infiltration of quacks and tradesmen who are not properly trained into the building industry in this country. In fact, Akindoyeni, Chinwokwu and Ogunsemi alluded that the use of substandard construction materials and poor workmanship were responsible for over 36% of the cases of building collapse in Nigeria [54]. It was on this premise that Chinwokwu insisted that the inability of the relevant bodies to ascertain the quality of construction materials and skill base of construction workers in the Nigeria building industry will continue to result into building collapse [22]. These issues are all centered on the inability of town planning and physical development control agencies to effectively deliver on their supervisory and inspection role in the building supply chain in the study area in particular and Nigeria as a whole.

## 7 Conclusions and recommendations

In line with the aim of this research which is to investigate the role town planning and approval authorities play in building collapse, the conclusion from the result of the study shows that the weakest links in the building chain and construction industry leading to the cause of incessant building collapse are.

• inadequate staffing of qualified professionals in the local town planning and approval offices
• Non-monitoring compliance with approved plans, environment laws, existing codes, standards and non-enforcement of violations of these parameters; the deficient monitoring and supervisory responsibility.
• Incomplete document requirements usually requested by town planning and approval authority and
• Insufficient assessment checklist for the town planning office use to vet construction drawings before granting approval for site work.

This lies all within the purview of the government and its statutory regulatory agencies. It was discovered that the government major concern and interest is in fees for physical planning approval and sundry levies without adequate supervision of the building project. With the enormous magnitude of human and material loss as evident in the study associated with building collapse, it is important for the government to urgently review policy guidelines and staffing to make Nigeria to curb incessant building collapse in the country. However, if no visible practical action is taken to regulate the business of construction and diligently prosecute defaulters and withdraw ownership of erring property, the implication is that incidences of collapsed buildings will continue to rise unabated with it concomitant effects on the social, economic and environmental landscape of this country. The government through the Town Planning Authorities in collaboration with building professionals are advised to do the following to curb incessant building collapse in Nigeria.

• Adequate number of qualified building professionals should be employed by government to man sensitive positions in Town Planning and Approval Authorities.
• Government should take proactive steps by empowering Town Planning Authorities to discharge their functions of strict enforcement of physical development bye-laws and pieces of regulation by providing them the necessary logistic such as patrol vans and other equipment.
• In view of reported ineptitude and corruption among the staff of Town Planning and Approval Authorities in the cause of discharging their duties, it is suggested that condition of services of this calibre of works be improved with competitive wages and compensations so that they can resist the temptations of unethical practices. However, there is also a need to sanction erring officers found wanting in the discharge of their duties.
• There is also a need for Town Planning and Approval Authorities to review their list of documents required for building approval to include soil tests and services drawings and others as stipulated in the 2006 National Building Code. This is to ensure that all critical aspects of the building plan approval process are given equal attention they deserve.

Although this studies can be considered to have achieved its purpose, it is not without some limitations. First, the study is limited to Enugu Metropolis, southeast Nigeria; and thus the findings cannot be generalised for all cities and towns in Nigeria. Consequently, it is recommended that further studies be conducted in other parts of Nigeria. Second, the research design adopted was survey, which relied on questionnaire, interview guide and photographic materials as the principal data collection instruments; and thus the findings are limited to the bias of the participants. In view of this, further studies are suggested using experimental design and numerical simulation. Third, the study focused on the role of Town Planning and Approval Authorities in Enugu Metropolis, meaning that several other aspects of the cause of building collapse were not included in the current study. Other studies are therefore suggested to investigate the roles of other stakeholders in the building supply chain and possible include more participants for a different perspective on this subject matter.

## Declarations

### Author contribution statement

Francis Okeke: Conceived and designed the experiments; Performed the experiments; Analyzed and interpreted the data; Contributed reagents, materials, analysis tools or data; Wrote the paper.

Chinwe Sam-Amobi: Performed the experiments; Contributed reagents, materials, analysis tools or data.

### Funding statement

This research did not receive any specific grant from funding agencies in the public, commercial, or not-for-profit sectors.

### Competing interest statement

The authors declare no conflict of interest.

### Additional information

No additional information is available for this paper.
